# The Multi-Sensor and Multi-Temporal Dataset of Multiple Crops for In-Field Phenotyping and Monitoring

**DOI:** 10.1038/s41597-025-06462-y

**Published:** 2026-01-08

**Authors:** Yue Linn Chong, Julie Krämer, Erekle Chakhvashvili, Elias Marks, Felix Esser, Ansgar Dreier, Radu Alexandru Rosu, Kevin Warstat, Ralf Pude, Sven Behnke, Onno Muller, Uwe Rascher, Heiner Kuhlmann, Cyrill Stachniss, Jens Behley, Lasse Klingbeil

**Affiliations:** 1 Center for Robotics, University of Bonn, 53115 Bonn, Germany; 2 Institute of Bio- and Geosciences, IBG-2: Plant Sciences, Forschungszentrum Jülich GmbH, 52428 Jülich, Germany; 3 Institute of Geodesy and Geoinformation, University of Bonn, 53115 Bonn, Germany; 4 Department for Computer Science, University of Bonn, 53115 Bonn, Germany; 5 Institute of Crop Science and Resource Conservation, Faculty of Agricultural, Nutritional and Engineering Sciences, University of Bonn, Bonn, Germany; 6 Field Lab Campus Klein-Altendorf, Faculty of Agricultural, Nutritional and Engineering Sciences, University of Bonn, Bonn, Germany; 7 Lamarr Institute for Machine Learning and Artificial Intelligence, Bonn & Dortmund, Germany

**Keywords:** Agriculture, Plant sciences, Scientific data

## Abstract

Phenotyping is crucial for understanding crop trait variation and advancing research, but is currently limited by expensive, labor-intensive monitoring. New phenotypic trait monitoring methods are being proposed to reduce this so-called phenotyping bottleneck via automation. These methods are often data-driven, requiring a dataset recorded with a specific sensor and corresponding reference values for developing novel methods. To this end, we present the MuST-C (Multi-Sensor, multi-Temporal, multiple Crops) dataset, which contains field data from various sensors collected over a growing season, covering six crop species. All data was georeferenced for alignment across sensors and dates. To collect our dataset, we deployed aerial and ground robotic platforms equipped with RGB cameras, LiDARs, and multispectral cameras, aiming to capture a wide variety of modalities and observations from different viewpoints. In addition to sensor data, we also provide manually collected leaf area index and biomass reference measurements. Our dataset enables the development of novel automatic phenotypic trait estimation methods, allows comparisons across different sensors, and generalizability across crop species.

## Background & Summary

Agricultural systems need to meet the demands of a growing population while coping with climate change^1,2^. The efficiency of sustainable agricultural systems can be increased through research and development of crop varieties that can both provide high yields and cope with climate impacts^3,4^. Breeding such crop varieties and investigating innovative management methods requires assessing traits based on phenotypic measurements^5^. However, phenotyping is a time-consuming and laborious task that is still often performed manually using destructive measurements. The frequency of manual measurements is limited by labor costs, and the destructive nature of these measurements further increases resource requirements, as substantial plant material and plot area are needed. These limitations restrict the rate at which measurements can be generated, leading to slower breeding decisions^3^.

Moving towards high-throughput phenotyping of plants by automation^6–9^ is, therefore, a key stepping stone towards high temporal frequency, repeatability, and objectiveness of measurements for phenotypic trial experiments. The development of innovative high-throughput phenotyping methods using mobile sensing is enabled by the availability of domain-specific data under real-world conditions. While some real-world agricultural field datasets are available^10–12^, these datasets often only represent a single growth stage, crop, or sensor type and are not developed for reusability. Thus, currently, we cannot directly compare different approaches that use different sensor modalities, study the effects of various growth stages on the performance of developed methods, or investigate the capabilities of developed approaches on multiple crops or sensors.

**Fig. 1:**
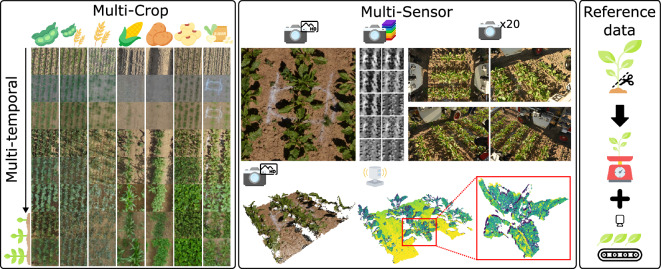
Overview of our dataset, comprising a field trial with multiple crops, with sensor data collected at several time points. We collected data using multiple sensors; here, we showcase our georeferenced data, showing the same location in a sugar beet plot across multiple sensors in the middle section. From top left, we show the data from RGB orthophotos, multispectral orthophotos, four of the 20 RGB instantaneously captured images, a colored dense point cloud from RGB structure-from-motion, and the high-resolution LiDAR point cloud (highlighted in red, we showcase the detailed point cloud of a single plant). With our georeferenced data, we can align data from different sensors and dates. In addition, we provide destructive reference measurements for aboveground fresh weight and LAI for the field trials.

We fill this gap by providing a multi-sensor, multi-temporal, multi-crop (MuST-C) dataset (c.f. Fig. 1) to support research in high-throughput phenotyping by accelerating the development of algorithmic approaches for phenotypic trait estimation. To this end, we performed measurements using different sensing modalities, including RGB cameras, multispectral cameras, and range measurements from light detection and ranging (LiDAR) sensors, over multiple growth stages on a field trial with multiple crops. We mounted the sensors on robotic platforms, i.e., unmanned aerial vehicles (UAVs) and unmanned ground vehicles (UGVs), equipped with global navigation satellite system (GNSS) receivers for georeferencing, which enabled us to provide the data in the same reference frame such that all data is aligned across sensors and over time. To evaluate newly developed approaches, we provide reference data acquired with conventional measurements of plant traits, specifically, the biomass and the leaf area index (LAI). We measured the LAI using a hand-held commercial canopy analyzer (i.e., SunScan canopy analysis system), and validated the measurements with destructive measurements using the WinDIAS leaf area meter.

In addition to LAI^13,14^ and biomass estimation^15^, our MuST-C dataset is useful for addressing other tasks, such as crop-weed segmentation^16^, plant counting^16,17^, leaf counting^18,19^, vegetation index retrieval, plant height^20^, and leaf angle distribution^7^. Furthermore, additional tasks that our dataset supports include plant reconstruction^21^, plant density estimation^22^, vegetation segmentation^23,24^, or radiation use efficiency estimation^3^. In line with growing interest in foundation models^25–28^, our data can be used for self-supervised pretraining foundation models for agricultural applications, such as weed semantic segmentation^9^, disease detection^29^, or 3D reconstruction^12^. Our dataset provides the novelty of aligned data from multiple sensors, possibly for comparison and development of novel methods from different sensor modalities, including sensor fusion approaches.

## Methods

### Experimental Design

**Fig. 2:**
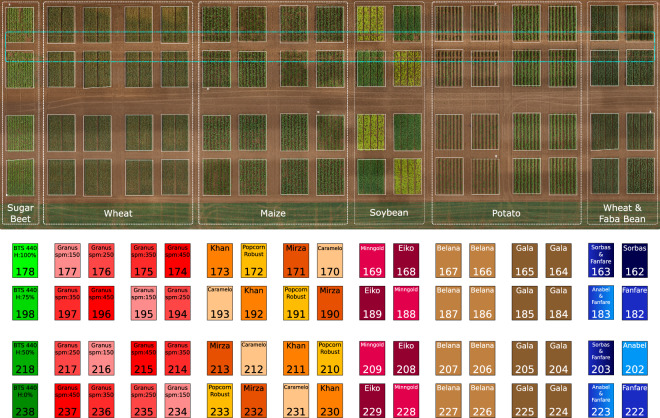
Orthophoto of the field trial area (top) and field trial layout (bottom). In the orthophoto, we denote the area of each species with white dashed lines. We designated an area for destructive measurements, shown here in light blue. In the field layout, we show the plot ID (bottom) and crop genotype (top) for each plot. We color-coded the plots based on the crops with sugar beets in green, wheat in red, maize in orange, soybean in pink, potato in brown, and intercrops in blue. For sugar beet plots, we mark the herbicide levels. For the wheat plots, we mark the seed density in seeds per square meter.

Figure 2 shows an orthophoto of the field trial, located at the Campus Klein-Altendorf research facility, University of Bonn, Germany ($5{0}^{\circ }3{7}^{{\prime} }$ North, ${6}^{\circ }5{9}^{{\prime} }$ East). The study site soil was classified as Haplic Luvisol, with a loamy siltic texture, and had high nitrogen levels (100 kg total available Nitrogen per hectare) in the plow layer, which decreased in the subsoil. The crops were cultivated in rectangular plots of 7.5 m by 6 m. The field experiment spanned one growing season in the spring of 2023; Table 1 shows the sowing and harvest dates for each crop. We report weather data collected from a Campbell Scientific environmental station equipped with a CS310 quantum sensor, a ClimaVUE50 weather sensor, and a CS655 soil moisture sensor (Campbell Scientific, Logan, UT, USA).

**Table 1:** Sowing dates and harvest dates of each species.

Species	Sowing Date (week #)	Harvest Date (week #)
Sugar beet	05.05.2023 (18)	N.A.
Wheat	25.04.2023 (17)	23.08.2023 (34)
Maize	18.05.2023 (20)	16.10.2023 (42)
Soybean	05.05.2023 (18)	11.10.2023 (41)
Potato	19.05.2023 (20)	27.09.2023 (39)
Wheat and faba bean intercrop	02.05.2023 – 03.05.2023 (18)	22.08.2023 – 22.08.2023 (34)

We harvested the sugar beets at a later date after the duration of this dataset’s development.

Our multi-crop field trial consists of six sub-experiments, comprising five monocultures: (i) sugar beet (*Beta vulgaris* L.), (ii) spring wheat (*Triticum aestivum* L.), (iii) sweet corn (*Zea mays* L.), (iv) soybean (*Glycine max* L.), and (v) potato (*Solanum tuberosum* L.), as well as a wheat-faba bean (*Vicia faba* L.) intercrop experiment, i.e., where wheat and faba bean crops are sown in the same plot.

In the sugar beet experiment, we tested different herbicide concentrations applied on the variety BTS 440: (i) 0% (no herbicide), (ii) 50%, (iii) 75%, and (iv) 100% of the amount used following standard agricultural practice of herbicide application. Sugar beet plants were sown on different dates for each column of plots to study the earlier stages of development. In the wheat experiment, we tested the wheat of the Granus variety with four sowing densities: (i) 150 seeds m^−2^, (ii) 250 seeds m^−2^, (iii) 350 seeds m^−2^, and (iv) 450 seeds m^−2^. The experiment followed a randomized complete block design with four replicates (*n* = 4) allocated to the plots. In the sweet corn experiment, we tested four varieties (Khan, Popcorn Robust, Mirza, and Caramelo), while in the soybean experiment, we tested two varieties (Eiko and Minngold). Both experiments followed a randomized complete block design with the replication *n* = 4 allocated to the plots. We planted two varieties of potatoes (Belana and Gala) using a two-block design. We managed the five monoculture trials conventionally, following standard agricultural practice in the region. In the wheat-faba bean trial, we compared organically grown intercrops with their respective monocultures as controls. The monocropped wheat was sown at 320 seeds m^−2^ and monocropped faba bean were sown at 36 seeds m^−2^. The intercrops were established as a 1:1 cereal-legume mixture using half of the monocrop sowing density per crop. We tested two mixtures: (i) Fanfare (faba bean) with short-growing Anabel (spring wheat) and (ii) Fanfare with tall-growing Sorbas (summer wheat). No fertilizers or herbicides were applied in this trial to mimic organic farming practices. The experiment was arranged in a two-block design: one block contained the mixture treatments (each with two replicates), while the second block was assigned to the mono-crop treatments (two replicates for faba bean and no replication for spring wheat).

### UAV and UGV Data Acquisition and Processing

We collected data from the aforementioned field experiment using different sensor modalities equipped on three different UAVs (UAV1, UAV2, and UAV3) and a UGV. Table 2 shows a complete summary of our data products, Table 3 shows the platforms used in our measurement campaign, and Table 4 shows the weeks and crops for each data package. In the following, we describe the different sensors and the collected data.

**Table 2:** The data types in our dataset.

Sensor	Data Package	Sensor Name	Sensor Manufacturer	Products	Resolution
RGB Camera (high resolution)	**UAV1-RGB**	PhaseOne iXM-100 RGB camera	PhaseOne, Copenhagen, Denmark	images, orthophotos, point clouds	11664 × 8750 px; 10^5^ pts m^−2^
LiDAR	**UAV2-LIDAR**	RIEGL miniVUX-SYS	RIEGL Laser Measurement Systems GmbH, Horn, Austria	point clouds	10^3^ pts m^−2^
RGB	**UAV2-RGB**	Sony *α* 7R	Sony, Tokyo, Japan	images, orthophotos	17320 × 6046 px
Multispectral	**UAV3-MS**	RedEdge-MX Dual Camera System	AgEagle Sensor Systems Inc., Wichita, KS, USA	images, orthophotos	1280 × 960 px
RGB	**UAV3-RGB**	Sony *α* 7R	Sony, Tokyo, Japan	images, orthophotos	6240 × 4160 px
RGB (20 cameras)	**UGV-RGB**	Nikon Z7 (20 cameras)	Nikon Corporation, Tokyo, Japan	images	8256 × 5504 px
Laser Triangulation Scanner	**UGV-LMI**	LMI Gocator 2490 laser triangulation scanners	LMI Technologies, Burnaby, Canada	point clouds	10^6^ pts m^−2^
LiDAR	**UGV-Ouster**	Ouster OS1	Ouster, Inc., San Francisco, CA, USA	point clouds	10^4^ pts m^−2^
Non-destructive LAI	**md_SunScan**	SunScan Plant canopy analyzer	Delta-T Devices Ltd., Cambridge, UK	LAI	N/A
Destructive LAI	**md_Destructive_LAI**	WinDIAS Leaf Image Analysis System	Delta-T Devices Ltd., Cambridge, UK	LAI	N/A
Biomass	**md_Biomass**	N/A	N/A	Aboveground fresh weight	N/A

We collected five modalities with robotic platforms and manually collected LAI and biomass reference measurements. We collected all the data in the same field trial. We report the image resolution for RGB and multispectral sensors and report the approximate points per square meter for the point clouds provided.

**Table 3:** UAVs and UGVs used for data collection.

Platform	UAV1	UAV2	UAV3	UGV
Name	DJI Matrice 300 RTK (M300)	DJI Matrice 600 PRO (M600)	DJI Matrice 600 PRO (M600)	Thorvald II + custom aluminum housing
Manufacturer	SZ DJI Technology Co., Ltd., Shenzhen, China	SZ DJI Technology Co., Ltd., Shenzhen, China	SZ DJI Technology Co., Ltd., Shenzhen, China	Saga Robotics, Oslo, Norway
GNSS	RTK (built-in)	RTK (built-in) + Applanix APX-20 IMU + Applanix AV14 GNSS	RTK (built-in)	multi-GNSS and SBG Ellipse D IMU
Altitude	21 m	30 m	25 m	N/A
Flight plan	lawnmower pattern (perpendicular to the field plots)	cross-flight pattern (15 m side distance)	lawnmower pattern (parallel to the Sun’s trajectory)	N/A

Further details about the UGV can be found in our prior publication^31^.

**Table 4:** Calendar weeks where we collected data for each data package and crop species.

	

Legend: : Sugar Beet, : Wheat, : Maize, : Soybean, : Potato, : Intercrop.

#### UAV1 High-Resolution RGB Images (**UAV1-RGB**)

**Fig. 3:**
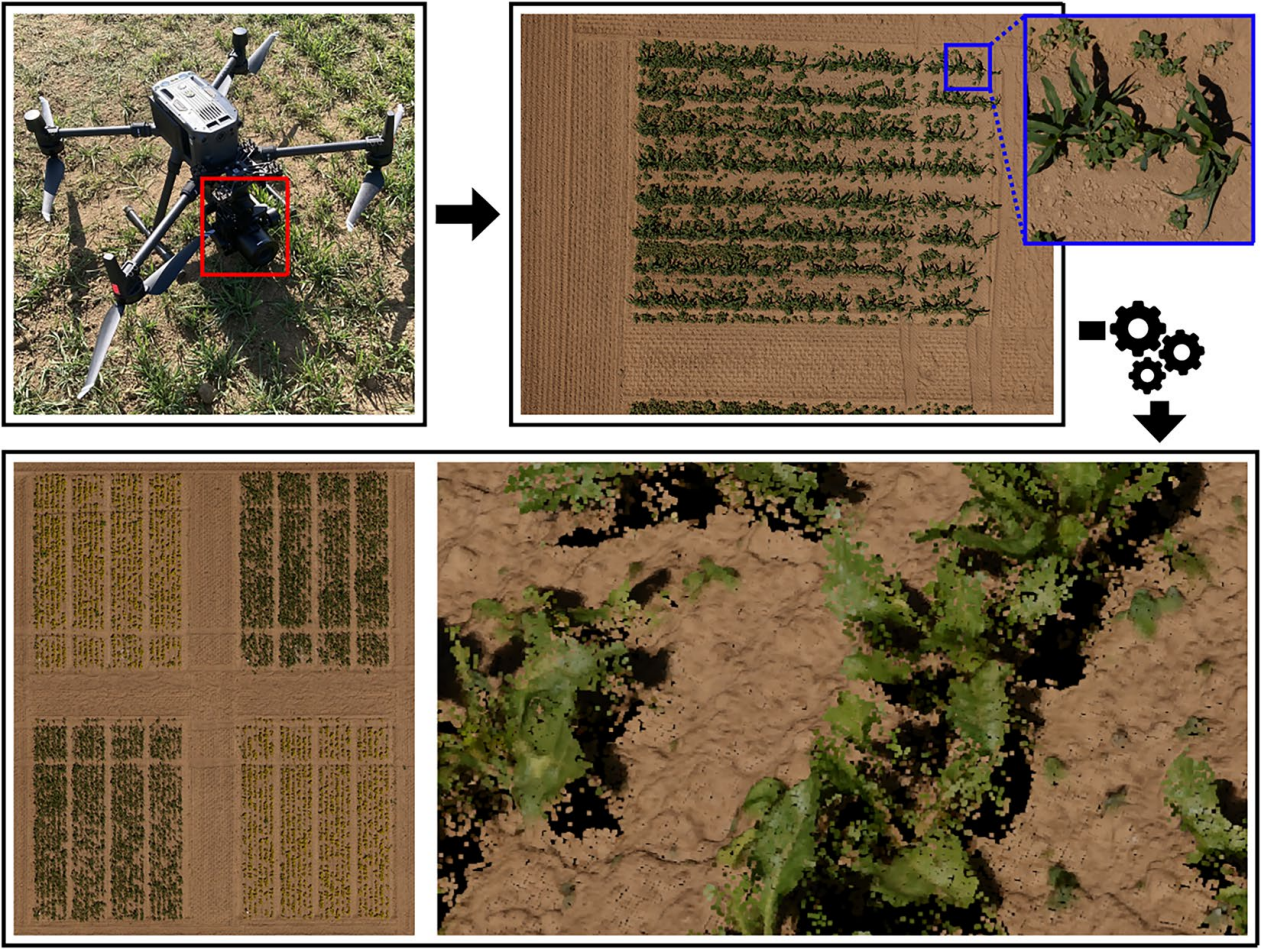
From the top left, the UAV setup consisting of the M300 with the PhaseOne camera (highlighted in red), which captured high-resolution images; the images have a large field of view, yet the small details are also visible (in blue). We performed structure from motion using these high-resolution RGB images and obtained a dense point cloud and an orthophoto.

Figure 3 shows the UAV setup and products for **UAV1-RGB**. We collected high-resolution RGB images (11664 × 8750 pixels) in nadir view, i.e., where the camera points directly below, perpendicular to the ground. The captured images had a 70% overlap on the sides and a 74% overlap frontally. We flew UAV1 at an altitude of 21m, to obtain images with a ground sampling distance of approximately 1 mm. Using the Agisoft Metashape Pro software package, we performed structure from motion via bundle adjustment initialized with RTK poses, aligned using ground control points (GCPs), and obtained a photogrammetric point cloud and an orthophoto for each collection date.

#### UAV2 LiDAR Point Clouds (**UAV2-Lidar**)

**Fig. 4:**
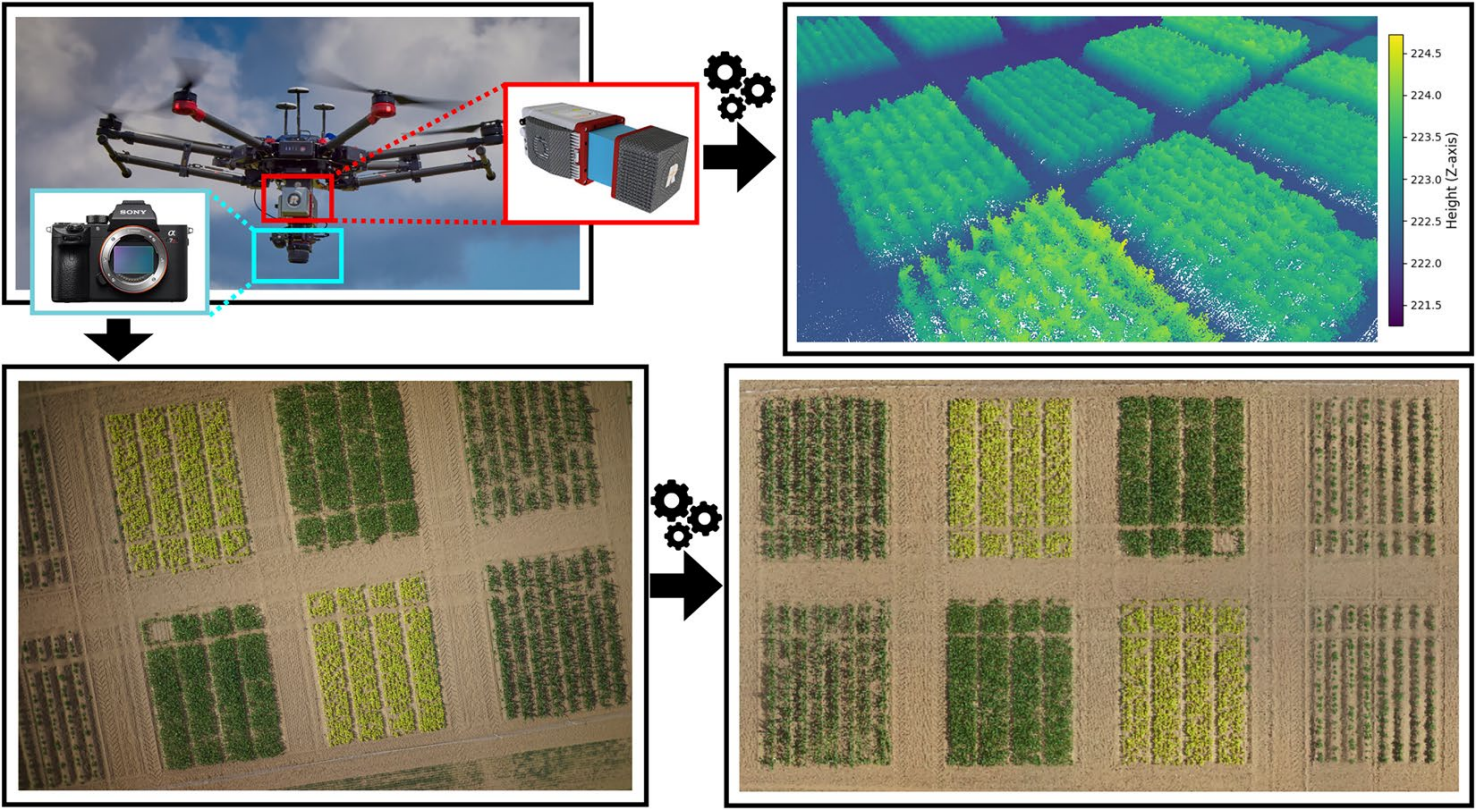
From top left, the UAV setup of the UAV2 with the RIEGL LiDAR (highlighted in red) and Sony RGB camera (highlighted in blue). We processed the data to obtain point clouds and orthophotos of the field.

Figure 4 shows the UAV setup and products for **UAV2-Lidar**. We outfitted the UAV2 with a RIEGL miniVUX-SYS consisting of the RIEGL miniVUX-2UAV 2D laser scanner and the Applanix APX-20 inertial measurement unit (1mm) (Applanix, Richmond Hill, Ontario, Canada). We configured the LiDAR sensor to the laser pulse repetition rate of 200 kHz and the scan speed of 53.80 lines per second. For georeferencing, we used a reference station to estimate the GNSS baseline and performed pose estimation using the Applanix POSPac software with the IMU and additional GNSS data. We used the software RIEGL RiPROCESS for direct georeferencing, which combined the trajectory and LiDAR data, with strip adjustment correction to improve the trajectory by using multiple flight strips. We filtered the LiDAR data by the maximum range of 45 m, and the resulting georeferenced point cloud has a mean density of 1433 pts m^−2^.

#### UAV2 RGB Images (**UAV2-RGB**)

In addition to the LiDAR data, we used the same UAV2 to simultaneously capture RGB data at 1.5 s intervals. We processed the images using the Agisoft Metashape Pro to obtain the orthophoto of the field for each date, an example of which is shown in Fig. 4. The orthophotos have a ground sampling distance of approximately 1 cm.

#### UAV3 Multispectral Images (**UAV3-MS**)

**Fig. 5:**
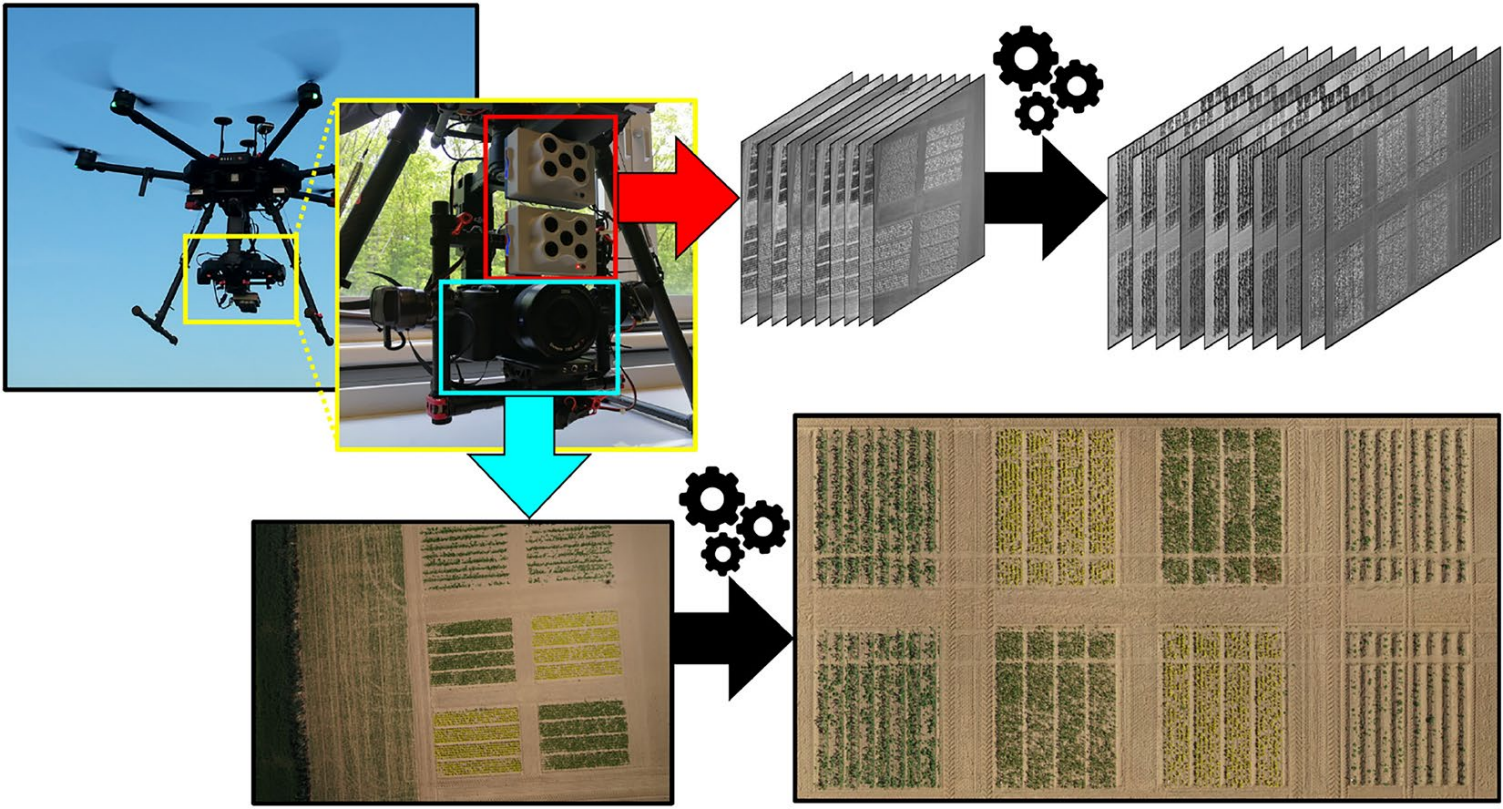
From top left, the UAV3 used to carry multispectral RedEdge cameras (in red box), which captured multispectral images (top middle). We processed the multispectral images to obtain orthophotos of the field trial with 10 reflectance channels. The same UAV also carried an RGB camera (highlighted in light blue), which captured RGB images during the same flight. We performed structure from motion with these RGB images to obtain an RGB orthophoto for each collection date.

We collected multispectral images using UAV3, mounted with the multispectral cameras with 10 bands, detailed in Table 5. The cameras point directly below, perpendicular to the ground, using the Ronin MX gimbal (SZ DJI Technology Co., Ltd., Shenzhen, China) to acquire images of the entire field trial with a ground sampling distance of 3 cm. Figure 5 shows the UAV setup and products for **UAV3-MS**. The UAV followed a lawnmower pattern, with an overlap of 90 % along the flight path and 65 % on the sides. To ensure valid orthophotos, we planned the flight campaigns on days with stable illumination conditions.

**Table 5:** Multispectral bands of the RedEdge-MX Dual Camera System used for **UAV3-MS** and their corresponding bands in our reflectance orthophotos.

Orthophoto Band	Name	Center Wavelength (nm)	Bandwidth (nm)
01	Costal blue	444	28
02	Blue	475	32
03	Green 1	531	14
04	Green 2	560	27
05	Red 1	650	16
06	Red 2	668	14
07	Red Edge 1	705	10
08	Red Edge 2	717	12
09	Red Edge 3	740	18
10	Near-infrared (NIR)	842	57

We calibrated each orthophoto’s reflectance with nine calibration panels with near-Lambertian surfaces and contrasting reflectance factors ranging from black to white, placed on the ground before each flight. Each panel has precisely defined, measurable reflectance values ranging from 2% to 63% reflectance. We placed the nine panels with some distance apart to avoid adjacency effects of the panels (neighboring panels contaminating each other’s reflectance). We put them on the bare soil to prevent the influence of the vegetation. We processed the raw multispectral images to radiance units^30^ and georeferenced them using ground control points using the Agisoft Metashape Pro software package to obtain the camera extrinsic calibration and multispectral orthophotos with the same resolution as the raw images. For accurate georeferencing, we recalibrated the camera poses using GCPs evenly distributed in the field trial. The exact location of each GCP was measured using the GNSS Receiver HiPer VR (Topcon Positioning Systems, Inc., Tokyo, Japan), with a relative base error of 5 mm horizontally and 10 mm vertically; and additional distance-dependent error of 0.5 parts per million horizontally and 0.8 parts per million vertically. We calibrated the multispectral reflectance orthophotos using the empirical line method^30^. In summary, **UAV3-MS** contains the multispectral images, camera calibrations, and multispectral reflectance orthophotos of the entire field for each collection date.

#### UAV3 RGB Images (**UAV3-RGB**)

In addition to the multispectral data of **UAV3-MS**, we also recorded RGB data during each UAV3 flight. We mounted an RGB camera on the same gimbal as the RedEdge and captured RGB images in sync with the multispectral images. From these RGB images, we obtained digital elevation models (DEMs) and orthophotos using the Agisoft Metashape Pro. The orthophotos have a ground sampling distance of approximately 3 cm, with an estimated overlap of approximately 80 % along the flight path and 75 % to the sides. In summary, **UAV3-RGB** contains the RGB images, RGB camera calibrations, RGB orthophotos, and DEMs for each collection date (Fig. 5).

#### UGV RGB Multi-Cameras (**UGV-RGB**)

**Fig. 6:**
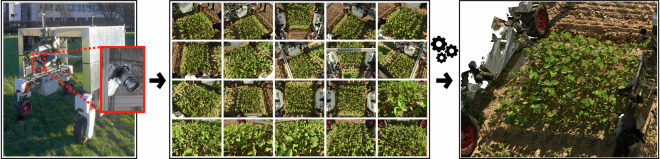
This figure shows the UGV (left) with the mounted cameras (one highlighted in red). There are 20 cameras mounted in the UGV, simultaneously capturing 20 images (middle). We used these images to perform structure from motion and obtained a 3D representation of the captured area (right).

Our UGV was equipped with 20 RGB cameras to capture images instantaneously, at the resolution of 45.7MP (8256 × 5504 pixels). We strategically positioned the cameras along the interior surfaces of the UGV and adjusted their zoom levels to capture a larger crop area. A translucent plastic sheet was attached to the sides of the UGV to minimize motion artifacts by shielding the plants from wind and reducing harsh shadows caused by bright sunlight. The UGV followed the path along the area of destructive sampling shown in Fig. 2, and captured images of the field trial. We collected the multi-camera data at the lower growth stages and stopped when the canopy was too tall or crowded. For each day, we manually calibrated each camera’s zoom level based on the crop canopy height. Esser *et al*.^31^ described the sensor setup in more detail. We sorted the sets of images based on the field plots. We calibrated the camera intrinsic and scaled extrinsics of each day using the Agisoft Metashape Pro. In summary, the **UGV-RGB** data package contains the sets of instantaneously captured 20 RGB images organized by plots and camera calibrations for each collection date. Figure 6 shows the UGV and cameras, and an example set of 20 instantaneously captured images, which we used to generate a mesh.

#### UGV LiDAR Point Clouds (**UGV-LMI** and **UGV-Ouster**)

We mounted two LMI laser triangulation scanners and an Ouster OS1 64-beam LiDAR on our UGV. The two triangulation scanners, mounted on the left and right sides of the UGV, had an overlapping field of view, and the multi-beam Ouster LiDAR at the front of the UGV was angled downward. We drove the UGV across all the plots in the field trial along the area of destructive sampling shown in Fig. 2.

For georeferencing, we interpolated the trajectory with respect to the laser data and transformed each laser profile from the scanners into a global coordinate frame (UTM, WGS84). We performed the system calibration (i.e., the estimated transformation between the scanners and the inertial navigation system’s frame) using a 3D point feature approach^32^. With the calibrations, for each LiDAR (left LMI, right LMI, and Ouster), the sequences of point clouds were respectively merged into a single large point cloud, which was then cropped and organized by plot.

Each 3D point in the point clouds contains time (UTC) and laser intensity ranging from 0 to 255. The point clouds from the left and right LMI scanners were not aligned to each other in the raw point clouds, and some misalignment between the two sensors may occur due to deformation (<10 cm) of the UGV when traversing uneven or soft ground. Therefore, we performed post-processing to merge the point clouds from the left and right LMIs using a custom plane-to-plane iterative closest point algorithm to obtain the final merged point clouds.

**Fig. 7:**
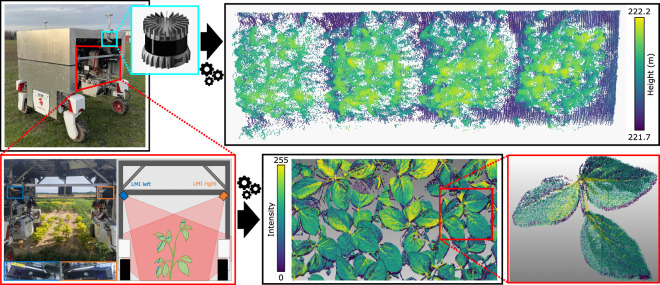
From top right, the UGV equipped with the Ouster LiDAR (highlighted in light blue), which we used to obtain a point cloud (top right). We also mounted the UGV with two LMI scanners, on the left and right (bottom left); the left LMI is highlighted in blue and the right LMI in orange. We mounted the two LMIs to have an overlapping field of view over the crop plants. We processed the LMI outputs to obtain a georeferenced dense point cloud (bottom middle). The point clouds have a high resolution, and individual plant organs are visible (bottom right, soy bean leaves manually cropped out).

Figure 7 shows the UGV setup and laser sensors used, as well as an example of an LMI point cloud (left and right scanner combined) for a soybean plot, and an example of the Ouster point cloud for a soybean plot; notice that the LMI point cloud has a higher density compared to the Ouster, where single plant organs are distinguishable with more detail. In short, **UGV-LMI** and **UGV-Ouster** respectively comprise one cropped point cloud for each plot and collection date.

### SunScan LAI Data Acquisition and Processing

**Fig. 8:**
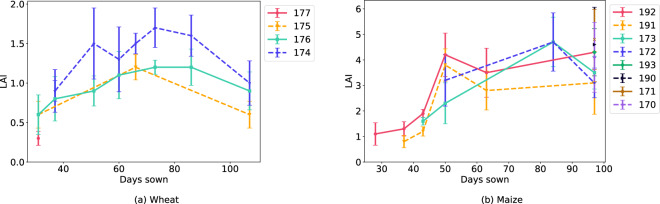
Example LAI from SunScan against the number of days since sowing and plot IDs of wheat and maize. The error bars for the SunScan measurements indicate the minimum and maximum measurements.

We took non-destructive LAI measurements with the SunScan Canopy Analysis System on the dates indicated in Table 4. All measurements were taken during midday (approximately between 12:00 and 15:30 local time), under mostly sunny conditions, and were repeated 10 times for the plots of sugar beet, wheat, maize, soybean, and wheat-faba bean intercrop trials of plots given in Table 6. The SunScan system consisted of an incoming light sensor and a probe. The incoming light sensor measured both direct and diffuse incoming radiation above the canopy and was placed on a tripod near the measurement area. The probe had 64 photosynthetically active radiation (PAR) sensors. We placed the probe diagonally below the canopy and spanning across multiple crop rows, in the center of the plot, to measure the under-canopy PAR. From the incoming light sensor and probe measurements, we calculated the LAI with the SunScan proprietary software, where we set the estimated leaf angle distribution parameter (ELADP) based on the crop species, as shown in Table 6. Figure 8 shows the plots of the LAI of each crop species over time.

**Table 6:** The plot IDs where we took SunScan measurements, the ELADP used to calculate LAI for SunScan, plot IDs where we took destructive LAI measurements, and plot IDs where we took destructive biomass measurements.

Species	SunScan plot IDs	ELADP	Destructive LAI plot IDs	Biomass plot IDs
Sugar beet	178, 179, 198, 199	1.55	178, 198	178, 198
Wheat	174 – 177	0.96	174, 176	174, 176
Maize	170 – 173, 190 – 193	1.37	191, 192	191, 192, 172, 173
Soybean	168, 169, 188, 189	4.1	168, 169, 188, 189	168, 169, 188, 189
Intercrop	162, 163, 182, 183	0.96	162, 163, 182, 183	162, 163, 182, 183

### Destructive Biomass and LAI Data Acquisition and Processing

We measured the aboveground fresh weight of plants sampled destructively from the field trial from the designated area shown in Fig. 2, on dates indicated in Table 4, for selected plots listed in Table 6. We harvested the length of *l*_*s*_, of *r*_*s*_ ∈ {4, 5} rows of wheat and intercrops, respectively, and an area of approximately 1 m^2^ for all other crops to obtain *N*_*s*_ plant individuals. We weighed each sugar beet and maize plant individually to record the biomass per plant. We avoided harvesting plants around the plot edges to minimize edge effects, and weighed the plants immediately after harvesting to obtain the biomass measurement *m*. Finally, we calculated the fresh weight of the aboveground biomass per meter *m*_crop_ (in g/m^−2^) of the plot with:

1 $${m}_{crop}=\{\begin{array}{ll}\frac{{r}_{m}}{{r}_{s}{l}_{s}}m, & for\,crop\in \{wheat,intercrop\}\\ \frac{{N}_{m}}{{N}_{s}}m, & for\,crop\in \{sugar\,beet,maize,soybean\}\end{array},$$

where *r*_*m*_ is the number of rows per meter of the plot, and *N*_*m*_ is the average number of plants per meter of the plot.

**Fig. 9:**
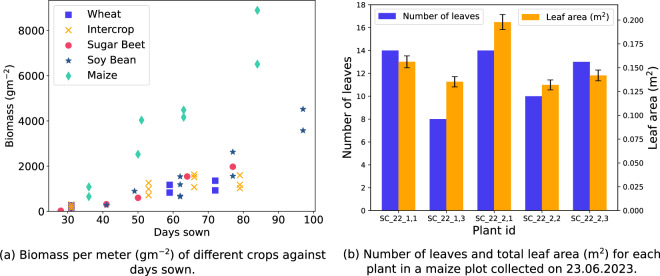
Example plots of biomass and LAI from destructive measurements. In (**a**), we show the range of biomass measurements obtained for multiple crops at various growth stages. In (**b**), we showcase the individual plant-level data from destructive measurements.

In addition, we measured the destructive leaf area using the WinDIAS 3 image analysis system leaf scanner. These destructively obtained LAI measurements were used to validate the SunScan LAI measurements (see Technical Validation section). On dates indicated in Table 4 and for selected plots listed in Table 6, after measuring the biomass of the harvested samples, we separated a subsample of the plants to scan for leaf area. From these plants, we detached all leaves from the stems and scanned each leaf using the WinDIAS 3 leaf scanner, with an overhead camera that takes images at 4 Hz to obtain total leaf area *A*. We scanned each sugar beet and maize plant individually to record the leaf area per plant. We scaled the total leaf area *A* to estimate the LAI of the plot, where LAI is the unitless ratio of measured green leaf area in square meters to one square meter of ground surface^33^ (m^2^ m^−2^) with:

2 $${LAI}_{crop}=\{\begin{array}{ll}\frac{{r}_{m}}{{r}_{s}{l}_{s}}\frac{m}{{m}_{w}}A, & for\,crop\in \{wheat,intercrop\}\\ \frac{{N}_{m}}{{N}_{s}}A, & for\,crop\in \{sugar\,beet,maize,soybean\}\end{array},$$

where *m*_*w*_ is the biomass of the plants subsampled and scanned for LAI, *l*_*s*_ is the length of the rows harvested in meters. The destructive leaf area measurements have an accuracy of ±4%, as reported by the WinDIAS manufacturer, but due to the scaling in Eq. (2), the calculated LAI is expected to have a poorer accuracy. Figure 9 shows example graphical plots of the destructively measured biomass and LAI.

## Data Records

**Fig. 10:**
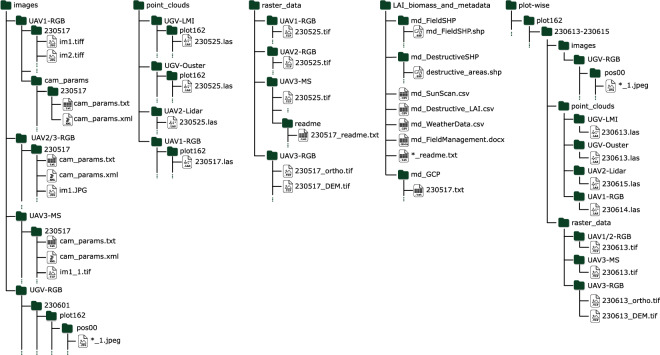
Directory tree structure of our dataset. We provide our data in two alternative organizational structures for the user to choose from. Firstly, we categorized all data packages according to their data type at the top level, i.e., images for raw image data, point_clouds for 3D data points, raster_data for orthophotos, and LAI_biomass_and_metadata for reference measurements and field data. Secondly, we provide an alternative organization of our data in the “plot-wise” directory for use cases that require plot-level analysis.

The MuST-C dataset is available via our project webpage https://www.ipb.uni-bonn.de/data/MuST-C/or directly via the bonndata public access repository 10.60507/FK2/OX9XTM^34^. Figure 10 illustrates the directory tree structure of our data records. We provide our data in two formats to support different use cases. Firstly, we organized the data into four directories according to their data types: (i) images, (ii) point clouds, (iii) raster data, and (iv) reference measurements and metadata. Secondly, for users interested in plot-level data, we provide our data organized by plot in the “plot-wise” directory. Note that the information in both structures is duplicated; the users are free to choose the organization structure that best suits their use case.

The images directory consists of RGB and multispectral image files from **UAV1-RGB,** **UAV2-RGB,** **UAV3-RGB,** **UAV3-MS**, and **UGV-RGB**, with a child directory for each data package and a child directory for each date. For **UGV-RGB**, we organized the data into directories based on plots, and we sorted each set of 20 images into a directory, with each image filename following the nomenclature “nikon_*<camera ID>*.jpeg”. For the multispectral images of **UAV3-MS**, image filenames follow the nomenclature “IMG_*<image ID>*_*<band ID>*.tif”, following band IDs in Table 5. All camera calibrations were provided in the same directory as the images, in two alternative plain text formats, “cam_params.txt” and “cam_params.xml”.

The point clouds directory includes the LiDAR data (**UAV2-Lidar,** **UGV-LMI**, and **UGV-Ouster**) and the structure-from-motion dense point cloud of RGB images (**UAV1-RGB**). For **UAV1-RGB**, we provide the dense point cloud from bundle adjustment for each data collection date, which we cropped to follow the plots, resulting in a point cloud for each plot and date. For **UAV2-Lidar**, there is one cumulative point cloud of the whole field trial for each date, named “*<YYMMDD>*.las”. For **UGV-LMI** and **UGV-Ouster**, the point clouds are cropped to follow the plots, with the filenames of **UGV-LMI** merged point cloud files named according to their collection date “*<YYMMDD>*.las”. Similarly, for **UGV-Ouster**, the point cloud filenames follow the naming convention “*<YYMMDD>*.las”. The UGV point clouds are organized into directories by plot and by date.

Orthophotos from **UAV1-RGB,** **UAV2-RGB,** **UAV3-MS**, and **UAV3-RGB** are included in the raster data directory, with a child directory for each data package. For **UAV1-RGB** and **UAV2-RGB**, each orthophoto shows that the entire field trial for a given date is stored in a *.tif file format and named “*<YYMMDD>*_ortho.tif” For **UAV3-MS**, each multispectral reflectance orthophoto was named “*<YYMMDD>*.tif”; refer Table 5 for the details of each band. For **UAV3-RGB**, in addition to the orthophotos, we also provide the DEM files following the filename nomenclature “*<YYMMDD>*_DEM.tif”.

The LAI_biomass_and_metadata directory contains metadata and reference measurements. **md_FieldManagement** reports the field management notes, **md_FieldSHP** contains the shapefiles with details on plot-level variabilities, and **md_WeatherData** includes data from the weather station. We also report measurements taken on the field, including LAI measurements from the SunScan in **md_SunScan**, destructively measured LAI of each plot and leaf area per plant for sugar beets and maize in **md_Destructive_LAI**, fresh weight of the aboveground biomass for each plot (gm^−2^) and for individual plants of sugar beets and maize (g) in **md_Biomass**, and GCP locations in **md_GCP**. The areas where we took the destructive measurements are denoted in the metadata **md_DestructiveSHP**. We included further metadata of images **UAV3-MS,** **UGV-RGB**, shapefiles (**md_FieldSHP** and **md_DestructiveSHP**), and csv files (**md_Biomass,** **md_Destructive_LAI,** **md_SunScan**, and **md_WeatherData**) in readme files located in their respective directories.

In the plot-wise directory, we provide a duplicate copy of our data, organized by plot, allowing users to download sensor data for specific plots of interest. The data of each plot is further organized by dates, with data from similar dates collated together. Under this organizational structure, we performed additional post-processing to crop out each plot area, providing plot-wise data. We also performed further post-processing on the point clouds of **UAV2-Lidar** and **UGV-Ouster** with statistical outlier removal and manual segmentation. This enables users interested in plot-wise data to directly use our dataset without any further processing.

To further improve the usability of our large dataset, we also provide a sample of our data for download, which includes all LAI and biomass measurements, as well as data from a single plot (plot ID 198) for a single time point (~14.06.2023) for all data packages. Additionally, we also provide a sample of plot-wise data of the aforementioned date and plot. To assist with downloading our dataset, we provide a user interface on our website at https://www.ipb.uni-bonn.de/data/MuST-C/as well as plain text files containing the file URLs for automated downloads in our GitHub repository.

## Technical Validation

### Sensor Data and Georeferencing

**Fig. 11:**
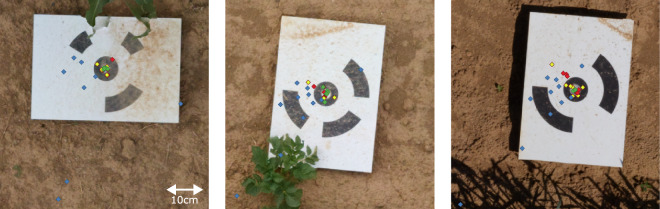
GCP locations of **UAV1-RGB** (green), **UAV2-RGB** (blue), **UAV3-RGB** (yellow), and **UAV3-MS** (red), for each data collection date where the GCPs are visible. The GCP locations in the orthophotos are mostly aligned across sensors and different collection dates.

We verified the quality of our dataset’s georeferencing of raster orthophotos and point clouds. For the orthophotos, we validated the georeferencing across time as well as across sensors (**UAV1-RGB,** **UAV2-RGB,** **UAV3-RGB**, and **UAV3-MS**) by checking the consistency of the GCP locations. To this end, we selected three GCPs, and selected at least three orthophotos from each sensor where the GCPs are visible. We manually marked the location of each GCP in each orthophoto from all sensors, shown in Fig. 11, indicating that the alignment of the GCP locations in most orthophotos across sensors and different collection dates is within the expected range. For each sensor, the location error, i.e., mean distance to the actual GCP location, is 0.7 cm for **UAV1-RGB**, 13.0 cm for **UAV2-RGB**, 2.3 cm for **UAV3-RGB**, and 1.9 cm for **UAV3-MS**. The error for **UAV2-RGB** is higher than that of other sensors because GCPs were not used for their structure from motion optimisation. Overall, the standard deviation of each GCP is at the centimeter level, which is sufficient for most phenotyping use cases.

**Fig. 12:**
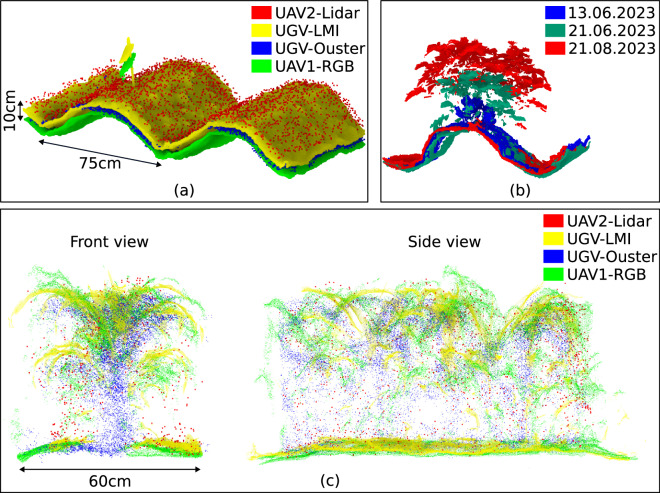
Point clouds of the potato plots (**a**) across different sensors and (**b**) over time. In (**a**), when the potatoes were just sown, we show the point clouds from **UAV2-Lidar** in red, **UGV-LMI** in yellow, **UGV-Ouster** in blue, and **UAV1-RGB** in green. The alignment in X and Y direction is almost perfect, with the undulation of the soil (75 cm row width) and the experiment tagging stick. Between the point clouds, there is a small discrepancy of less than 10 cm in the Z direction. In (**b**), we show the point clouds from **UGV-LMI** for the same potato plant across three dates (13.06., 21.06., and 21.08.) to show the alignment over time with our georeferenced sensor data. In (**c**), we show the point clouds of three maize plants from the front (along a row) and the side. The clouds have been generated from different sensor data, taken on the same day. The variations in density and distribution of points between sensors are shown here.

For the point clouds, Fig. 12(a) illustrates the alignment of the point clouds from different sensors and shows that the point clouds are well aligned, indicating the validity of each georeferenced point cloud. The stick with the vertical experiment tag used to mark plots is fully aligned in all point clouds, with less than 10 cm discrepancy mostly along the z-axis. In Fig. 12(b), we show the good alignment of the point clouds across time, and similarly in Fig. 12(c), we show the good alignment of point clouds across sensors. In addition, the georeferencing accuracy of the **UAV2-Lidar** system has been investigated in our prior work^35^. For **UGV-LMI** and **UGV-Ouster**, we registered and georeferenced the point clouds by fusing the SBG IMU and GNSS position and heading data using a graph-based pose optimization approach as described in our prior work^31^, which results in a georeferencing accuracy of about 1-2 cm, where the repeatability of a single LMI laser line measurement is reported by LMI Technologies by 12 micrometers (https://lmi3d.com/wp-content/uploads/2020/02/DATASHEET_Gocator_2490_US_WEB.pdf) and the Ouster OS1 range precision is specified with 1 cm at a range from 1-20 m (https://data.ouster.io/downloads/datasheets/datasheet-revd-v2p0-os1.pdf).

### LAI Reference Measurements

**Fig. 13:**
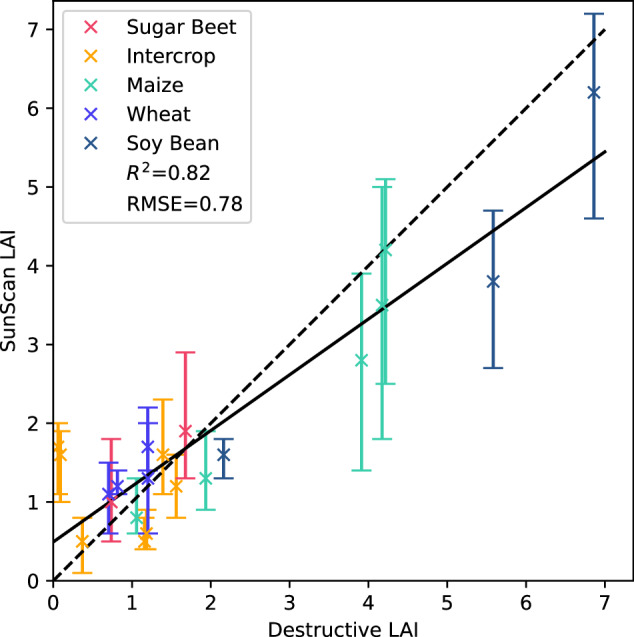
SunScan validation plot of LAI measured using SunScan against LAI measured with destructive measurements, with RMSE of 0.78 and R^2^ of 0.82. The error bars for the SunScan measurements indicate the minimum and maximum measurements.

We validated the LAI measurements from the SunScan with destructive measurements. Figure 13 plots the LAI measured using SunScan and destructive measurements on similar dates, i.e., at most one day apart, to show the correlation between the two data types with the R^2^ of 0.82 and root mean square error (RMSE) of 0.78. Where available, we report the LAI of the area where crops were sown; see the readme of **md_SunScan** for further details. The correlation plot indicates a strong agreement between destructive measurements and SunScan measurements.

However, the SunScan and destructive LAI measurements have some discrepancies, likely due to the following points. Firstly, the plots were heterogeneous, particularly in maize and soybeans, leading to a high variance in the SunScan measurements and deviation between SunScan and destructive measurements. We took multiple repeated measurements in the plot with SunScan at different locations to fully capture the heterogeneity of the plot, but destructive measurements were taken only from one area per sample, which may not fully reflect the whole plot. We marked the location where each destructive measurement was taken in a shapefile **md_DestructiveSHP** for future LAI retrieval usage.

Secondly, SunScan measures LAI with PAR sensors and does not distinguish between green and brown vegetation, while destructive measurements only measure the green parts of leaves. This difference in measurements leads to higher SunScan measurements for the later developmental stages of wheat, both in single crop plots and intercrop plots, as the wheat senesces and sheds its lower leaves (heading and ripening).

## Usage Notes

The MuST-C dataset can be used to estimate phenotypic traits, and here we illustrate this with an example use case for LAI estimation. Moreover, our dataset comprises data from multiple sensors, where we can obtain different phenotypic traits from each sensor using state-of-the-art methods. Here, we leverage RGB images for crop-weed segmentation, plant instance counting, and leaf detection, multispectral reflectance orthophotos for vegetation indices extraction, and point clouds for plant height and leaf angle estimation. While these methods leverage the strengths of each sensor, our dataset is aligned over time and across sensor modalities so that we can transform and merge these extracted traits from one sensor to another.

### LAI Estimation

**Fig. 14:**
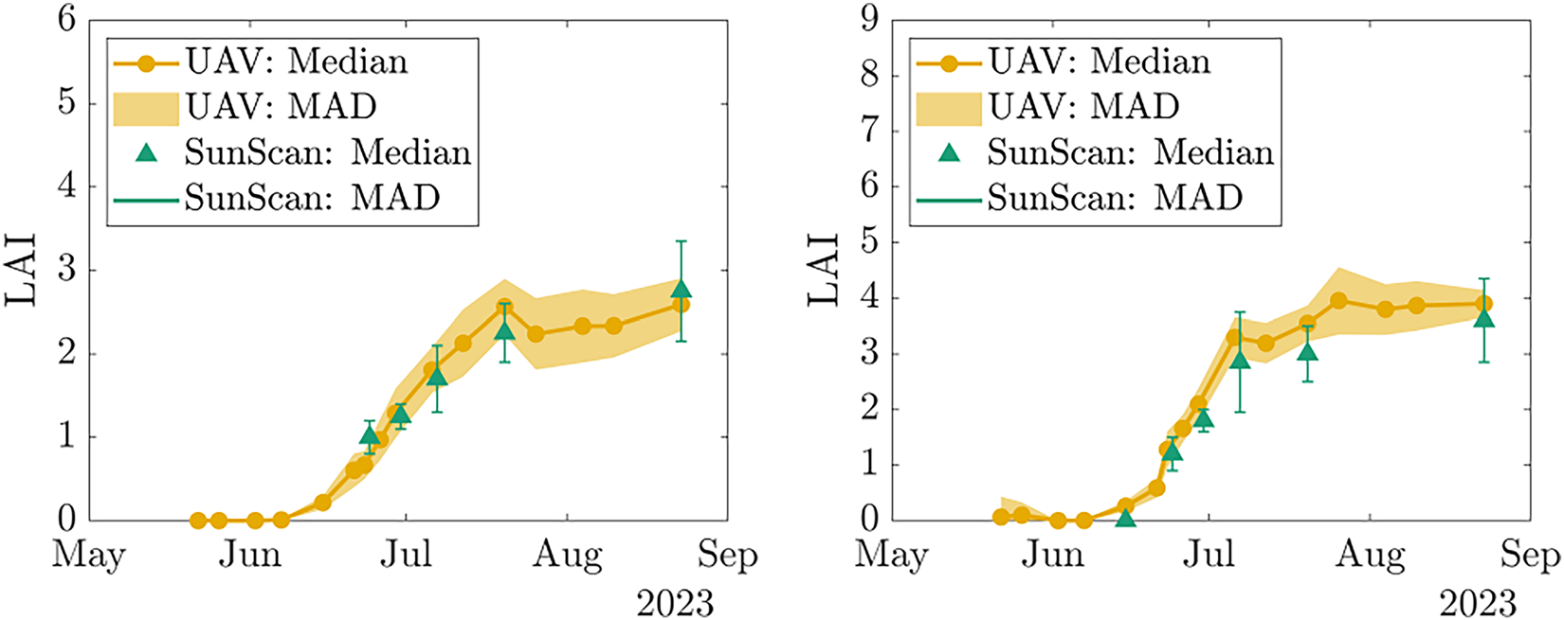
Example results of LAI from **UAV2-Lidar** data over several dates. We show the median and Mean Absolute Deviation (MAD) of the LAI estimated from **UAV2-Lidar** data and reference measurements from SunScan for maize (left) and sugar beet (right).

In our dataset, we measured the LAI values using a SunScan sensor and destructive sampling (Fig. 13). One of the use cases for this data set is to non-destructively derive LAI data from other sensor modalities. We exemplify this approach in the derivation of LAI from UAV Lidar data using Beer’s Law^36^. We used ray tracing to derive the probability of a laser ray being absorbed within the canopy and linked this to the LAI of the crop. Figure 14 shows the result in comparison with the data from the SunScan sensor. Other potential methods include multi-view geometry approaches based on the images^10^ or direct modelling of the plant structure^7^.

### Plant Counting

**Fig. 15:**
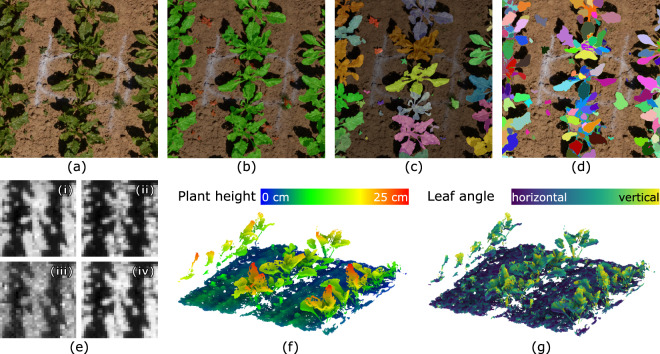
By leveraging the strengths of each sensor, our multi-sensor dataset enables several use cases such as (**b**) crop-weed semantic segmentation (**c**) plant count, and (**d**) leaf segmentation from RGB images (**a**),(**e**) vegetation indices ((i) NDVI, (ii) EVI, (iii) NDRE, and (iv) OSAVI) from multispectral images, and (**f**) plant height and (**g**) leaf angle from point clouds.

In addition to LAI estimation, several phenotypic traits and auxiliary information can be extracted using deep learning methods. Firstly, from the RGB images (e.g., Fig. 15(a)), we can obtain the plant count of a given plot using panoptic segmentation^37^ methods. Figure 15(b)(c) shows the results of a panoptic segmentation method^16^ trained on a labeled dataset^9^, on an RGB image from MuST-C’s **UAV1-RGB**. Qualitatively, the method can perform plant counting relatively well despite using weights trained on a different dataset.

### Crop Leaves Extraction

Several phenotypic traits and phenological development (expressed with the BBCH scale^38^) are related to the leaves of the crop, leaf length and width, and leaf angle. To assist in extracting these traits, we can first detect individual leaves of the crop. In Fig. 15(d), we show an example of instance segmentation^39^, which includes segments of individual leaf instances.

### Vegetation Indices

Vegetation indices are useful for use cases such as LAI estimation^14^, vegetation classification^40^, biomass estimation^41^, and so on. We can extract these vegetation indices using the multispectral and RGB products of our dataset. As an example, from the multispectral data of **UAV3-MS**, we extracted vegetation indices such as NDVI^42^, NDRE^43^, EVI^44^, and OSAVI^45^, shown in Fig. 15(e).

### Plant Height Extraction

The plant heights can be extracted from all point clouds by approximating a ground surface and calculating the differences to the ground (see Fig. 15(f)). To provide height values on plot level, mostly the mean of points above the 95th percentile of all height values in the crop for each plot is used^46^.

### Leaf Angle Estimation

The orientation of the leaf surfaces of plants is often referred to as ‘leaf angle distribution’ and determines how the canopy intercepts light. It is a crucial parameter in crop modelling and breeding, as it influences photosynthesis, biomass accumulation, and water-use efficiency. While there is no commonly used sensor to measure the leaf angles in field environments, the leaf angle distribution can be estimated using the high-resolution point clouds from the UGV-based triangulation scanners^7^. Figure 15(g) shows the local surface orientation for the points of some sugar beet plants.

## Data Availability

As mentioned in the section on Data Records, our data is available for download at our data repository 10.60507/FK2/OX9XTM^34^, which follows the nomencular conventions from Fig. 10.

## Code availability

The custom Python code for loading the MuST-C dataset, processing the leaf area destructive measurements, converting multispectral orthophotos, and plotting the graphs in Figs. 8, 9, 13 is made publicly available at https://github.com/PRBonn/MuST-C. We also provide a developer’s kit to process our dataset (e.g., to organize the data at the plot level) in the same GitHub repository.

## References

[1] Schmidhuber, J. & Tubiello, F. N. Global food security under climate change. *Proceedings of the National Academy of Sciences* **104**, 19703–19708, 10.1073/pnas.0701976104 (2007). 10.1073/pnas.0701976104 PMC2148361 18077404

[2] Wijerathna-Yapa, A. & Pathirana, R. Sustainable Agro-Food Systems for Addressing Climate Change and Food Security. *Agriculture* **12**, 1554, 10.3390/agriculture12101554 (2022).

[3] Reynolds, M. *et al*. Breeder friendly phenotyping. *Plant Science* **295**, 110396, 10.1016/j.plantsci.2019.110396 (2020). 32534615 10.1016/j.plantsci.2019.110396

[4] Watt, M. *et al*. Phenotyping: New Windows into the Plant for Breeders. *Annual Review of Plant Biology* **71**, 689–712, 10.1146/annurev-arplant-042916-041124 (2020). 32097567 10.1146/annurev-arplant-042916-041124

[5] Araus, J. L. & Cairns, J. E. Field high-throughput phenotyping: the new crop breeding frontier. *Trends Plant Sci.* **19**, 52–61, 10.1016/j.tplants.2013.09.008 (2014). 24139902 10.1016/j.tplants.2013.09.008

[6] Hennecke, F., Bömer, J. & Heim, R. H. Modification of an automated precision farming robot for high temporal resolution measurement of leaf angle dynamics using stereo vision. *MethodsX* **14**, 103169, 10.1016/j.mex.2025.103169 (2025). 39897648 10.1016/j.mex.2025.103169 PMC11787487

[7] Stausberg, L., Jost, B., Klingbeil, L. & Kuhlmann, H. A 3D Surface Reconstruction Pipeline for Plant Phenotyping. *Remote Sensing* **16**, 4720, 10.3390/rs16244720 (2024).

[8] Marks, E. *et al*. BonnBeetClouds3D: A Dataset Towards Point Cloud-Based Organ-Level Phenotyping of Sugar Beet Plants Under Real Field Conditions. In *Proc. of the IEEE/RSJ Intl. Conf. on Intelligent Robots and Systems (IROS)*, 10.1109/IROS58592.2024.10802820 (2024).

[9] Weyler, J. *et al*. PhenoBench — A Large Dataset and Benchmarks for Semantic Image Interpretation in the Agricultural Domain. *IEEE Trans. on Pattern Analysis and Machine Intelligence (T-PAMI)* **46**, 9583–9594, 10.1109/TPAMI.2024.3419548 (2024). 10.1109/TPAMI.2024.3419548 38923484

[10] Roth, L., Aasen, H., Walter, A. & Liebisch, F. Extracting leaf area index using viewing geometry effects-A new perspective on high-resolution unmanned aerial system photography. *ISPRS Journal of Photogrammetry and Remote Sensing* **141**, 161–175, 10.1016/j.isprsjprs.2018.04.012 (2018).

[11] Raj, R. *et al*. Leaf area index estimation using top-of-canopy airborne RGB images. *International Journal of Applied Earth Observation and Geoinformation* **96**, 102282, 10.1016/j.jag.2020.102282 (2021).

[12] Zermas, D., Morellas, V., Mulla, D. & Papanikolopoulos, N. Estimating the Leaf Area Index of Crops Through the Evaluation of 3D Models. In *Proc. of the IEEE/RSJ Intl. Conf. on Intelligent Robots and Systems (IROS)*, 10.1109/IROS.2017.8206517 (2017).

[13] Chakhvashvili, E. et al. LAI and Leaf Chlorophyll Content Retrieval Under Changing Spatial Scale Using a UAV-Mounted Multispectral Camera. In *Proc. of the IEEE Intl. Geoscience and Remote Sens. Symposium (IGARSS)*, 10.1109/IGARSS46834.2022.9883446 (2022).

[14] Shafian, S. *et al*. Unmanned aerial systems-based remote sensing for monitoring sorghum growth and development. *PLoS One* **13**, 1–15, 10.1371/journal.pone.0196605 (2018). 10.1371/journal.pone.0196605 PMC5929499 29715311

[15] Deery, D. M. *et al*. Ground-Based LiDAR Improves Phenotypic Repeatability of Above-Ground Biomass and Crop Growth Rate in Wheat. *Plant Phenomics* **2020**, 8329798, 10.34133/2020/8329798 (2020). 33313565 10.34133/2020/8329798 PMC7706344

[16] Weyler, J., Läbe, T., Behley, J. & Stachniss, C. Panoptic Segmentation with Partial Annotations for Agricultural Robots. *IEEE Robotics and Automation Letters (RA-L)* **9**, 1660–1667, 10.1109/LRA.2023.3346760 (2024).

[17] Fang, Y. *et al*. An automatic method for counting wheat tiller number in the field with terrestrial LiDAR. *Plant Methods* **16**, 132, 10.1186/s13007-020-00672-8 (2020). 33005214 10.1186/s13007-020-00672-8 PMC7526133

[18] Roggiolani, G. et al. Hierarchical Approach for Joint Semantic, Plant Instance, and Leaf Instance Segmentation in the Agricultural Domain. In *Proc. of the IEEE Intl. Conf. on Robotics & Automation (ICRA)*, 10.1109/ICRA48891.2023.10160918 (2023).

[19] Weyler, J., Magistri, F., Seitz, P., Behley, J. & Stachniss, C. In-Field Phenotyping Based on Crop Leaf and Plant Instance Segmentation. In *Proc. of the IEEE Winter Conf. on Applications of Computer Vision (WACV)*, 10.1109/WACV51458.2022.00302 (2022).

[20] Dreier, A., Lopez, G., Bajracharya, R., Kuhlmann, H. & Klingbeil, L. Structural wheat trait estimation using uav-based laser scanning data: Analysis of critical aspects and recommendations based on a case study. *Precision Agriculture* **26**, 18, 10.1007/s11119-024-10202-4 (2025).

[21] Marks, E., Magistri, F. & Stachniss, C. Precise 3D Reconstruction of Plants from UAV Imagery Combining Bundle Adjustment and Template Matching. In *Proc. of the IEEE Intl. Conf. on Robotics & Automation (ICRA)*, 10.1109/ICRA46639.2022.9811358 (2022).

[22] Wilke, N. *et al*. Assessment of plant density for barley and wheat using uav multispectral imagery for high-throughput field phenotyping. *Computers and Electronics in Agriculture* **189**, 106380, 10.1016/j.compag.2021.106380 (2021).

[23] Madec, S. *et al*. VegAnn, Vegetation Annotation of multi-crop RGB images acquired under diverse conditions for segmentation. *Scientific Data* **10**, 302, 10.1038/s41597-023-02098-y (2023). 37208401 10.1038/s41597-023-02098-y PMC10199053

[24] Marks, E. *et al*. High Precision Leaf Instance Segmentation in Point Clouds Obtained Under Real Field Conditions. *IEEE Robotics and Automation Letters (RA-L)* **8**, 4791–4798, 10.1109/LRA.2023.3288383 (2023).

[25] Stevens, S. et al. BioCLIP: A vision foundation model for the tree of life. In *Proc. of the IEEE/CVF Conf. on Computer Vision and Pattern Recognition (CVPR)*, 10.1109/CVPR52733.2024.01836 (2024).

[26] Gu, J. et al. Bioclip 2: Emergent properties from scaling hierarchical contrastive learning arXiv:2505.23883 (2025).

[27] Yang, C.-H. *et al*. BioTrove: A Large Curated Image Dataset Enabling AI for Biodiversity. In *Proc. of the Conf. on Neural Information Processing Systems (NeurIPS)* (2024).

[28] Nahian, M. J. A., Ghosh, T., Sheikhi, F. & Maleki, F. Agri-FM+: A Self-Supervised Foundation Model for Agricultural Vision. In *Proc. of the IEEE/CVF Conf. on Computer Vision and Pattern Recognition (CVPR) Workshops* (2025).

[29] Benfenati, A., Causin, P., Oberti, R. & Stefanello, G. Unsupervised deep learning techniques for automatic detection of plant diseases: reducing the need of manual labelling of plant images. *Journal of Mathematics in Industry* **13**, 5, 10.1186/s13362-023-00133-6 (2023).

[30] Chakhvashvili, E., Siegmann, B., Bendig, J. & Rascher, U. Comparison of reflectance calibration workflows for a UAV-mounted multi-camera array system. In *Proc. of the IEEE Intl. Geoscience and Remote Sens. Symposium (IGARSS)*, 10.1109/IGARSS47720.2021.9555143 (2021).

[31] Esser, F. *et al*. Field Robot for High-Throughput and High-Resolution 3D Plant Phenotyping: Towards Efficient and Sustainable Crop Production. *IEEE Robotics and Automation Magazine (RAM)* **30**, 20–29, 10.1109/MRA.2023.3321402 (2023).

[32] Esser, F., Tombrink, G., Cornelißen, A., Klingbeil, L. & Kuhlmann, H. System Calibration of a Field Phenotyping Robot with Multiple High-Precision Profile Laser Scanners. In *Proc. of the IEEE Intl. Conf. on Robotics & Automation (ICRA)*, 10.1109/ICRA57147.2024.10610208 (2024).

[33] Watson, D. J. Comparative Physiological Studies on the Growth of Field Crops: I. Variation in Net Assimilation Rate and Leaf Area between Species and Varieties, and within and between Years. *Annals of Botany* **11**, 41–76, 10.1093/oxfordjournals.aob.a083148 (1947).

[34] Chong, Y. L. et al. The multi-sensor and multi-temporal dataset of multiple crops for in-field phenotyping and monitoring. *BonnData*, 10.60507/FK2/OX9XTM (2025). 10.1038/s41597-025-06462-y PMC12796333 41507213

[35] Dreier, A., Janßen, J., Kuhlmann, H. & Klingbeil, L. Quality Analysis of Direct Georeferencing in Aspects of Absolute Accuracy and Precision for a UAV-Based Laser Scanning System. *Remote Sensing* **13**, 3564, 10.3390/rs13183564 (2021).

[36] Bailey, B. N. & Mahaffee, W. F. Rapid, high-resolution measurement of leaf area and leaf orientation using terrestrial LiDAR scanning data. *Measurement Science and Technology* **28**, 064006, 10.1088/1361-6501/aa5cfd (2017).

[37] Kirillov, A., He, K., Girshick, R., Rother, C. & Dollar, P. Panoptic segmentation. In *Proc. of the IEEE/CVF Conf. on Computer Vision and Pattern Recognition (CVPR)*, 10.1109/CVPR.2019.00963 (2019).

[38] Lancashire, P. D. *et al*. A uniform decimal code for growth stages of crops and weeds. *Annals of Applied Biology* **119**, 561–601, 10.1111/j.1744-7348.1991.tb04895.x (1991).

[39] Chong, Y. L. et al. Zero-Shot Semantic Segmentation for Robots in Agriculture. In *Proc. of the IEEE/RSJ Intl. Conf. on Intelligent Robots and Systems (IROS)* (2025).

[40] Milioto, A., Lottes, P. & Stachniss, C. Real-Time Semantic Segmentation of Crop and Weed for Precision Agriculture Robots Leveraging Background Knowledge in CNNs. In *Proc. of the IEEE Intl. Conf. on Robotics & Automation (ICRA)*, 10.1109/ICRA.2018.8460962 (2018).

[41] Banerjee, B. P., Spangenberg, G. & Kant, S. Fusion of spectral and structural information from aerial images for improved biomass estimation. *Remote Sensing* **12**, 3164, 10.3390/rs12193164 (2020).

[42] Rouse, J. Monitoring the vernal advancement and retrogradation of natural vegetation. *Tech. Rep.*, NASA Goddard Space Flight Center (1973).

[43] Barnes, E. et al. Coincident detection of crop water stress, nitrogen status and canopy density using ground based multispectral data. In *Proc. of the Conf. on Precision Agriculture* (2000).

[44] Huete, A. *et al*. Overview of the radiometric and biophysical performance of the MODIS vegetation indices. *Remote Sens. of Environment* **83**, 195–213, 10.1016/S0034-4257(02)00096-2 (2002).

[45] Rondeaux, G., Steven, M. & Baret, F. Optimization of soil-adjusted vegetation indices. *Remote Sens. of Environment* **55**, 95–107, 10.1016/0034-4257(95)00186-7 (1996).

[46] Hütt, C., Bolten, A., Hüging, H. & Bareth, G. UAV LiDAR metrics for monitoring crop height, biomass and nitrogen uptake: a case study on a winter wheat field trial. *Journal of Photogrammetry, Remote Sensing and Geoinformation Science* **91**, 65–76, 10.1007/s41064-022-00228-6 (2023).

